# Multi-Modal Representation via Contrastive Learning with Attention Bottleneck Fusion and Attentive Statistics Features

**DOI:** 10.3390/e25101421

**Published:** 2023-10-07

**Authors:** Qinglang Guo, Yong Liao, Zhe Li, Shenglin Liang

**Affiliations:** 1 School of Cyber Science and Technology, University of Science and Technology of China, Heifei 230027, China; 2 National Engineering Research Center for Public Safety Risk Perception and Control by Big Data (RPP), CETC Academy of Electronics and Information Technology Group Co., Ltd., China Academic of Electronics and Information Technology, Beijing 100041, China; 3 Department of Electrical and Electronic Engineering, The Hong Kong Polytechnic University, Hong Kong SAR, China; 4 School of Telecommunications Engineering, Xidian University, Xi’an 710071, China

**Keywords:** multimodal representation, contrastive learning, attention bottleneck fusion, attentive statistics features

## Abstract

The integration of information from multiple modalities is a highly active area of research. Previous techniques have predominantly focused on fusing shallow features or high-level representations generated by deep unimodal networks, which only capture a subset of the hierarchical relationships across modalities. However, previous methods are often limited to exploiting the fine-grained statistical features inherent in multimodal data. This paper proposes an approach that densely integrates representations by computing image features’ means and standard deviations. The global statistics of features afford a holistic perspective, capturing the overarching distribution and trends inherent in the data, thereby facilitating enhanced comprehension and characterization of multimodal data. We also leverage a Transformer-based fusion encoder to effectively capture global variations in multimodal features. To further enhance the learning process, we incorporate a contrastive loss function that encourages the discovery of shared information across different modalities. To validate the effectiveness of our approach, we conduct experiments on three widely used multimodal sentiment analysis datasets. The results demonstrate the efficacy of our proposed method, achieving significant performance improvements compared to existing approaches.

## 1. Introduction

Multi-modal fusion, which integrates information from multiple modalities into a compact and informative representation, poses a significant challenge as it requires effectively correlating the semantics of diverse modalities. In recent years, several approaches have been developed to learn the joint embeddings of multiple modalities [1,2]. However, each modality exhibits distinct representations and statistical features, making it difficult to capture complex intermodal correlations.

Deep learning techniques have demonstrated remarkable success in generating useful feature representations [3,4]. Consequently, these approaches learn a shared representation across the top layers of modality-specific networks, assuming that high-level representations contain sufficient semantic information and that common patterns across modalities exist at the semantic level.

However, there are two remaining issues with this method. Firstly, relying solely on high-level representations may not provide sufficient information. We employ a controlled cross-modal attention flow among the tokens within a layer to address this concern—namely, crafted features. Secondly, whether common patterns occur at the semantic level or a specific single layer of representation is unclear. In practice, fusion based on high-level representations functions similarly to traditional late fusion, which combines semantic notions from unimodal features. However, late fusion, in contrast to other fusion algorithms such as early fusion, can only capture connections at the semantic level and fails to harness other types of correlations, such as covariation at the early feature level [5] or hierarchical supervision over the entire network [6]. Consequently, statistics fusion is anticipated to capture the intricate relationships across modalities more effectively.

The ‘Early fusion’ model permits unrestrained attention flow across an image’s various spatial and temporal regions. Although theoretically promising, comprehensive pairwise attention across all model layers may be superfluous due to the high-density, fine-grained, yet largely redundant information within visual inputs. Additionally, such a model would struggle to effectively scale to longer videos, given the quadratic complexity of pairwise attention with token sequence length. To address these concerns, we employ a controlled cross-modal attention flow among the tokens within a layer. This is achieved by allowing unrestrained attention within a modality but obliging our model to gather and ‘condense’ information from each modality before exchanging it with another. At the heart of this proposal is introducing a limited number of latent fusion units, forming an ‘attention bottleneck.’ These units serve as mandatory conduits for all cross-modal interactions within a layer.

Contrastive learning has gained popularity as a paradigm for learning feature representations by solving an instance discrimination task [7,8,9]. Recent research has also explored its use for acquiring multimodal representations [10,11,12]. However, most of these studies focus on learning a cross-modal embedding space [10,11], aiming to identify knowledge transferred across modalities. Unfortunately, they do not explicitly investigate the fusion type of multiple modalities, thereby failing to exploit the synergistic potential of multimodal data fully.

**Figure 1:**
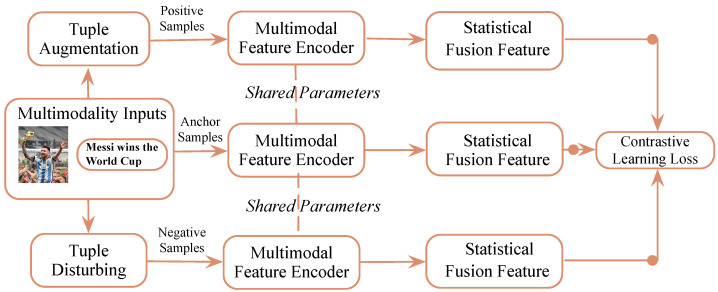
Illustration of our basic idea.

We propose a fusion approach called Attentive Statistics Fusion to address these issues. As shown in Figure 1, this approach incorporates significance-weighted standard deviations and weighted means for image features, leveraging an attention mechanism to assess their importance. By doing so, our method enables embeddings to more accurately and effectively capture multimodal elements with long-term fluctuations. Furthermore, we employ a Transformer-Encoder to combine the statistical modal features, allowing interactions among data vectors to be captured. This approach benefits from allocating greater attention weights to image patches and text tokens with explicit and latent associations, enabling the Transformer module to better align and fuse image and text features at the token level. As a multi-layer encoder, the Transformer-Encoder enhances the model’s abstraction capability and facilitates extracting deep features from multimodal input. To promote multimodal fusion explicitly, we apply a supervised contrastive loss (SupCon) specifically designed for this purpose. SupCon leverages positive samples created by enhancing anchors and utilizes hard negative samples with non-correspondent components. This ensures that the synergy between modalities and weak modalities is not overlooked.

The contributions of this paper can be summarized as follows:

- To address the ignorance of context statistics in the existing tensor-based fusion methods in image feature extraction, we propose statistics fusion, which correlates the features of different statistics features of images. Context statistics fusion provides a holistic perspective by integrating standard deviations and features. This enables embedding vectors to capture correlation variations efficiently and accurately.
- Attention bottlenecks are used to fuse statistical modal global features. Our model strategically curtails the cross-modal information flow between latent units via well-defined fusion ‘bottlenecks.’ These bottlenecks compel the model to collate and ‘condense’ the most pertinent inputs from each modality, ensuring that only the necessary information is shared with the other modalities. Multi-headed self-attention may assist in aligning and fusing token-level image and text features, which increases model abstraction capability.
- We aim for representation learning utilizing contrastive learning for multimodal data. The central concept is to compare multimodal anchor tuples with hard negative samples that disrupted modalities with improved positive samples acquired using an optimizable data augmentation procedure. Multiple positive samples are permitted per anchor via a supervised contrastive loss function.

## 2. Related Work

### 2.1. Multimodal Fusion

Extensive research has been conducted in multimodal fusion to explore diverse approaches to integrate and fuse information from multiple sensors, including images, videos, speech, and text.

One common strategy is feature-level fusion, where features from different sensors are extracted and combined to form a comprehensive representation [13,14]. This approach often utilizes traditional feature extraction algorithms such as Convolutional Neural Networks (CNNs) and Recurrent Neural Networks (RNNs) to extract useful features from image, audio, and textual data.

Another prevalent fusion strategy is decision-level fusion, where decisions or predictions from different sensors are combined to make a final decision [15,16]. Ensemble learning algorithms, such as voting or weighted voting, are commonly employed to integrate outputs from multiple sensors. Decision-level fusion techniques allow for combining the complementary strengths of different modalities to improve overall system performance.

Moreover, hybrid fusion techniques have also been explored, combining feature-level and decision-level fusion approaches [17,18,19]. These techniques aim to leverage the benefits of both strategies by fusing low-level sensory features and high-level decision outputs. Sophisticated algorithms, including deep neural networks and attention mechanisms, often employ hybrid fusion techniques to effectively integrate multimodal information at multiple levels.

### 2.2. Contrastive Learning

In recent years, numerous researchers have drawn their attention to contrastive Learning [20,21,22,23,24,25], owing to its extraordinary performance in sentiment analysis [26,27,28]. Many models, underpinned by contrastive learning, have been introduced in natural language processing and computer vision. Studies such as ConSERT [29], SimCSE [30], and CLEAR [31] demonstrate the applicability of contrastive learning within the sphere of natural language processing. MoCo [8], SimCLR [9], SimSiam [32], and CLIP [33] exhibit natural language processing’s deployment within the field of computer vision, showcasing considerable progress in zero-shot and few-shot learning.

More recently, contrastive learning has seen increasingly wide applications in multimodality. Huang et al. [34] leveraged intra-modal, inter-modal, and cross-lingual contrastive learning, significantly elevating video search performance. Yuan et al. [35] capitalized on the intrinsic data properties within each modality and cross-modal semantic information, enhancing the quality of learned visual representations.

In contrast with these works, we focus on aligning and fusing token-level features and learning their common sentiment-related features to elevate model performance further.

## 3. Methodology

To capture the correlation across different modalities more effectively, a commonly used approach is to directly concatenate the distinct characteristics of each modality and subsequently apply multiple layers of nonlinear transformations to construct a high-level joint representation [36]. This fusion technique is known as early multimodal fusion. However, it should be noted that while this concatenation-based fusion method adds dimension, it falls short in capturing intricate correlations that may exist across modalities [3].

To address the limitations of early multimodal fusion and better capture the complex correlations between modalities, a primary strategy involves reducing the impact of individual differences and emphasizing common meanings within the fused representation [4]. This is achieved by introducing a common layer at the center of the multimodal network, giving rise to what is known as intermediate multimodal fusion [37].

Building upon previous multimodal networks, it can be deduced that their fusion strategy typically involves incorporating one common layer alongside two modality-specific layers. These multimodal units effectively capture the correlations between different layers [3,38]. In our research, we adopt a dense multimodal fusion approach to uncover the intricate hierarchical relationships present within the representations of various modalities.

**Figure 2:**
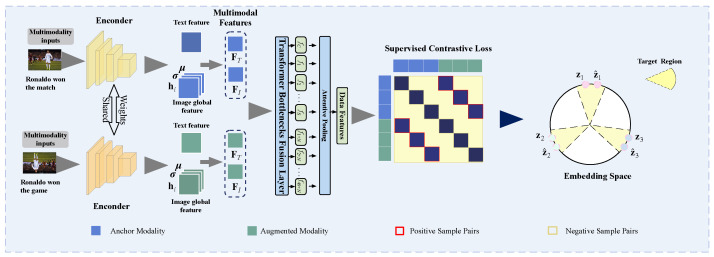
Our model leverages statistical features and Transformer for supervised contrastive learning. An embedding space is learned in which the same-sample pairs stay close to each other while different-sample pairs remain far apart.

To enhance the representations, we employ contrastive learning, which aims to maximize agreement across multiple enhanced views of the same data by utilizing a contrastive loss in the latent space. Our framework is illustrated in Figure 2.

### 3.1. Data Augmentation

To enhance the diversity and richness of our input samples, we apply random augmentation to each sample, resulting in a modified representation denoted as $\hat{x}=Aug\left(x\right)$. Each augmentation operation provides a distinct perspective and contributes a subset of the original sample’s information. Specifically, for image data, we employ a range of transformations such as cropping, rotation, contrast adjustment, inversion, flipping, solarization, posterization, brightness adjustment, and sharpness adjustment. On the other hand, for text data, we incorporate a random masking technique to introduce variability.

### 3.2. Encoder Network

Our objective is to train an encoder network denoted as $f_{\theta }\left(\cdot \right)$ using a set of labeled samples $X=\{x_{1},x_{2},\ldots ,x_{n}\}$. The role of $f_{\theta }\left(\cdot \right)$ is to transform each input text or image $x_{i}$ into an embedding vector $h_{i}=f_{\theta }\left(x_{i}\right)\in R^{d}$, where *d* represents the output dimension.

We employ the same encoder network for the original and augmented samples to achieve this, generating two separate representation vectors. Our approach uses BERT and ViT as encoders to extract hidden representations from the text and image inputs. These models are specifically chosen to capture the intricate features within the text data.

### 3.3. Channel Attention-Based Global Statistics Image Features

We compute the statistical properties of the extracted feature vectors to capture important characteristics. Specifically, we calculate the standard deviation and mean for each feature.

(1) $$\mu_{i}=\frac{1}{n}\sum_{j=1}^{n}h_{i}$$

(2) $$\sigma =\frac{1}{n}\sum_{j=1}^{n}{\left(h_{i}-\mu_{i}\right)}^{2}$$

(3) $$G_{I}=concat\left(h_{i},\mu ,\sigma \right)$$

where $h_{i}$ represents the *i*th image element and *n* denotes the number of samples. These global statistics features $G_{I}$ provide insights into the distribution and central tendency of the features, aiding in capturing salient information.

We introduce a channel attention mechanism to enhance the aggregated features’ representation power. This mechanism dynamically assigns weights to each channel in the aggregated feature vector, and the attention weight is calculated as follows:

(4) $$A=\mathrm{Softmax}(W_{g}\cdot \mathrm{ReLU}\left(W_{f}\cdot G_{I}\right))$$

where $W_{f}$ and $W_{g}$ are learnable weight matrices, Softmax represents the Softmax function, and ReLU denotes the rectified linear unit function.

The attention weights A reflect the importance of each channel in the aggregated feature vector $G_{I}$. By performing element-wise multiplication between the attention weights and the aggregated features, we obtain an attention-weighted feature vector:

(5) $$F_{I}=A⊙G_{I}$$

where ⊙ represents the element-wise multiplication operation. Incorporating the channel attention mechanism allows our model to focus on discriminative features while suppressing less informative ones, resulting in an enhanced feature representation for downstream tasks.

We consider the standard deviation a significant factor in our approach, encompassing the modal features related to long-term context variability. By incorporating the standard deviation, we aim to address the limitation of neural networks in fully capturing the expansive scope of information. While the vanishing gradient issue may restrict the network’s ability to comprehend global features effectively, introducing the standard deviation can help overcome this limitation by providing a measure of contextual distance and capturing broader, more encompassing features.

### 3.4. Multimodal Fusion via Transformer Bottlenecks

Human cognition seamlessly integrates high-dimensional inputs like sight and sound from multiple sources. In stark contrast, traditional machine perception models usually focus on single modalities optimized for unimodal benchmarks. Consequently, a prevalent approach for multimodal video classification is the ‘late-fusion’ technique, where each modality’s final representations or predictions are integrated later.

We employ a new Transformer-based architecture, using ‘fusion bottlenecks’ at multiple layers for modality integration. Unlike traditional pairwise self-attention, our model mandates that information from various modalities navigate through a limited number of latent bottlenecks. This strategy compels the model to consolidate and compress relevant data from each modality and disseminate only what is indispensable.

To mitigate the quadratic complexity inherent to pairwise attention, we incorporate a compact set of fusion bottleneck tokens, denoted as $Z_{\mathrm{fsn}}=\left[z_{\mathrm{fsn}}^{1},z_{\mathrm{fsn}}^{2},\ldots ,z_{\mathrm{fsn}}^{n}\right]$, into our input sequence. Consequently, the input sequence takes the form:

(6) $$F_{IT}=\left[F_{I}\left|Z_{\mathrm{fsn}}\right|F_{T}\right]$$

Our model is then designed to channel all cross-modal attention via these bottleneck tokens. To be precise, at layer l, token representations are calculated as follows:

(7) $$\overset{˜}{F}=\mathrm{Transformer}\left(\left[Z_{i}^{l}|Z_{\mathrm{fsn}}^{l}\right];\theta_{i}\right)$$

Here, Transformer refers to the Transformer-Encoder for multimodal data. z represents the fusion of text and image information. In essence, this process allows us to obtain a fused representation that encapsulates the combined characteristics of both text and image modalities. In this arrangement, we harness the potency of the Transformer for calculating the token representations, and the average of the new fusion bottleneck tokens is computed to update the fusion bottleneck for the next layer. The above measures streamline our model’s processing capabilities, leading to an overall enhancement in its performance.

### 3.5. Attentive Pooling

This work uses an attentive pooling mechanism to better capture the salient features in our input vector $\overset{˜}{F}$. Traditional pooling methods, such as max-pooling and average-pooling, often fail to consider the varying importance of elements in $\overset{˜}{F}$. Attentive pooling addresses this limitation by assigning learned attention scores to each element, creating a weighted input representation.

We first compute the attention scores α using a small neural network with parameters $W_{a}$ and $b_{a}$, followed by a softmax activation:

(8) $$\alpha =\mathrm{softmax}(W_{a}\overset{˜}{F}+b_{a})$$

(9) $$\alpha_{i}=\frac{\exp({\left(W_{a}\overset{˜}{F}+b_{a}\right)}_{i})}{\sum_{j=1}^{n}\exp\left({\left(W_{a}\overset{˜}{F}+b_{a}\right)}_{j}\right)}$$

The output o of the attentive pooling layer is then computed as the weighted sum of the input $\overset{˜}{F}$, weighted by the attention scores α:

(10) $$z=\sum_{i=1}^{n}\alpha_{i}\overset{˜}{F}$$

The model can focus on the most relevant elements in $\overset{˜}{F}$ for the task at hand through this attentive pooling mechanism.

### 3.6. Supervised Contrastive Losses

Supervised contrastive loss (SupCon) is utilized in scenarios where multiple samples with known labels belong to the same class. SupCon aims to enhance the discrimination of representations within the same class. The formulation of the SupCon loss is given by:

(11) $$L_{\mathrm{SupCon}}=\sum_{i=1}^{N}\frac{-1}{\left|P(i)\right|}\sum_{p\in P(i)}\log\frac{\exp(z_{i}\cdot z_{p}/\tau )}{\sum a\in A(i)\exp(z_{i}\cdot z_{a}/\tau )}$$

In Equation (11), P(i) represents the indices of positive samples within the augmented batch (consisting of both original and augmented samples) relative to the anchor $z_{i}$. |P(i)| denotes the cardinality of P(i). $z_{i}$ corresponds to the anchor sample, $z_{a}$ represents the negative samples, and $z_{p}$ denotes the positive samples. A(i) denotes the index set of negative samples.

The supervised contrastive loss can be combined with the cross-entropy loss as a form of regularization. The overall loss function is given by:

(12) $$\mathrm{Loss}=L_{\text{Cross-Entropy}}+L_{\mathrm{SupCon}}$$

By incorporating the SupCon loss alongside the cross-entropy loss, we aim to jointly optimize the model for both classification accuracy and enhanced discrimination within the same class.

## 4. Experiments and Results

### 4.1. Datasets

To evaluate the effectiveness of our proposed strategy, we conduct experiments on three publicly available datasets: MVSA-Single, MVSA-Multiple2 [39], and HFM3 [40]. These datasets are collected from Twitter and involve sentiment analysis tasks. Each text–image pair in these datasets is associated with a single sentiment label.

We preprocess the original MVSA datasets using the same procedure as [41] to ensure fair comparisons. Similarly, for the HFM dataset, we follow the preprocessing method outlined in [40]. This consistent preprocessing procedure ensures a standardized approach across all datasets.

For the MVSA datasets, we randomly split the data into training, validation, and test sets using an 8:1:1 ratio. This division allows us to effectively train and evaluate our models while reasonably balancing the datasets’ subsets.

Table 1 provides a detailed overview of the MVSA-Single, MVSA-Multiple, and HFM datasets. Interestingly, the HFM dataset is deployed as a binary classification mechanism within the multimodal sentiment analysis landscape. In contrast, the MVSA-Single and MVSA-Multiple are employed as ternary classification systems in the same domain.

### 4.2. Implementation Details

For our experiments, we utilize PyTorch and HuggingFace Transformers [42] libraries to implement both the baseline models and our proposed technique.

We employ the BERT-base as the text encoder within the fusion module and ViT [43] as the image encoder. These pre-trained models are chosen for their strong performance in capturing textual and visual features.

To specify the batch sizes for the experiments, we set them to 32 for MVSA-Single, 64 for MVSA-Multiple2, and 128 for HFM datasets. These batch sizes are selected to ensure efficient training while considering each dataset’s specific characteristics and computational requirements.

We use the AdamW optimizer with a learning rate of $2\times 10^{-5}$ to optimize the model parameters. This optimizer is well-suited for training deep neural networks and has shown effective performance in various natural language processing tasks.

All experiments are conducted on an A6000 GPU, which provides computational power for efficient model training and evaluation. The GPU accelerates the training process and enables faster experimentation.

### 4.3. Baselines

Our model is benchmarked against both unimodal sentiment models and multimodal baseline models.

**Unimodal Baselines:** We consider well-established models for text classification tasks like CNN, Bi-LSTM, and TGNN [44], a text-level graph neural network. BERT [45], a pre-trained model, is fine-tuned solely for text. For image modality, we have included OSDA [46], an image sentiment analysis model with multiple views, and ResNet [47], which is pre-trained and fine-tuned exclusively for images.

**Multimodal Baselines:** We compare our approach with several multimodal sentiment analysis models. These include MultiSentiNet [41], a deep semantic network with attention, and HSAN [48], a hierarchical semantic attentional network relying on image captions. Co-MN-Hop6 [49] is a co-memory network designed to model interactions across multiple modalities. MGNNS [50] integrates multi-channel graph neural networks with sentiment awareness for image-text sentiment detection. Schifanella et al. [51] propose a multimodal feature representation model that concatenates distinct feature vectors of different modalities; Concat(2) joins text and image features, while Concat(3) adds an extra image attribute feature. MMSD [40] offers a multimodal hierarchical fusion model blending text, image, and image attributes. Finally, Xu et al. [52] introduces the D& R Net, which builds the Decomposition and Relation Network to fuse text, image, and image attributes.

### 4.4. Results and Analysis

Table 2 compares the performance of our proposed model with benchmark methodologies. The evaluation metrics used for MVSA-Single and MVSA-Multiple2 are weighted-F1 and ACC, while for the HFM dataset, Macro-F1 and ACC are employed.

Our observations from the results are as follows: (1) Our model demonstrates comparable performance to other robust baseline models across all three datasets. (2) Our model outperforms the other baseline models on all three datasets. This could be attributed to the sparsity and noise present in the emotive characteristics of images, making it challenging for the models to extract meaningful features for sentiment analysis. In contrast, our approach incorporates statistical features of the images, while the multi-head attention method may capture the global features of the images. (3) The performance gain of the multimodal models is more limited for simpler tasks. For example, on the HFM dataset, the improvement in our model compared to BERT is less pronounced than on the MVSA-Single dataset. This can be attributed to HFM being a binary classification task, while MVSA-Single involves three-class classification.

Overall, our proposed model exhibits strong performance compared to the baseline models, indicating its effectiveness in capturing the sentiment information from both text and image modalities.

### 4.5. Ablation Study

We conducted further evaluations to assess the impact of the statistical fusion modules, Transformer-based multi-layer fusion modules, and supervised contrastive learning. The results of these experiments are presented in Table 3. The findings demonstrate that our model achieves the highest performance compared to all other models. This indicates that the multi-layer fusion module effectively fuses the multimodal data, improving performance.

Moreover, incorporating supervised contrastive learning enhances the model’s performance even further. This suggests that contrastive learning enables the model to learn common sentiment aspects while effectively differentiating between various sentiment data. By maximizing agreement and separation within the latent space, the contrastive learning mechanism aids in capturing essential patterns and improving the model’s ability to understand the underlying sentiment dynamics.

Overall, these results highlight the effectiveness of our proposed model, showcasing the benefits of the statistical fusion modules, Transformer-based multi-layer fusion modules, and supervised contrastive learning in capturing and leveraging multimodal sentiment information.

### 4.6. Compared with Funnel Transformer

**Figure 3:**
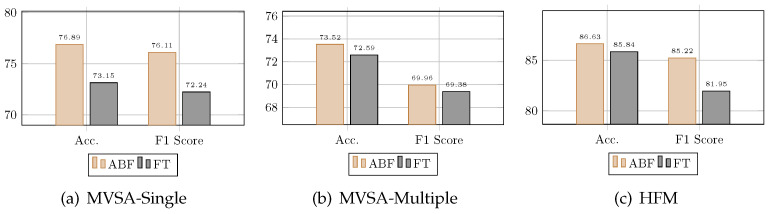
Comparative analysis of funnel Transformer fusion and attention bottleneck fusion. Attention bottleneck fusion model demonstrates superior performance in integrating text and image features. ABF refers to attention bottleneck fusion, while FT refers to funnel Transformer fusion.

The funnel Transformer [53] is an innovative modification of the standard Transformer architecture designed to enhance computational efficiency by filtering out sequential redundancy. A funnel-shaped encoder-decoder structure effectively compresses the input sequence while maintaining comparable performance across various natural language processing tasks. We replaced the attention bottleneck fusion module in our multimodal fusion framework with funnel Transformer fusion and conducted experiments on three datasets. As shown in Figure 3, our proposed model outperforms funnel Transformer fusion. We speculate that the reason for this is that the funnel Transformer is designed solely to reduce computational costs. In contrast, by introducing fusion bottleneck tokens and cross-attention mechanisms, attention bottlenecks selectively incorporate relevant portions from both text and images into the bottleneck tokens. This effective fusion of text and images is most beneficial for downstream sentiment analysis tasks.

### 4.7. The Effect of Transformer Layer

**Figure 4:**
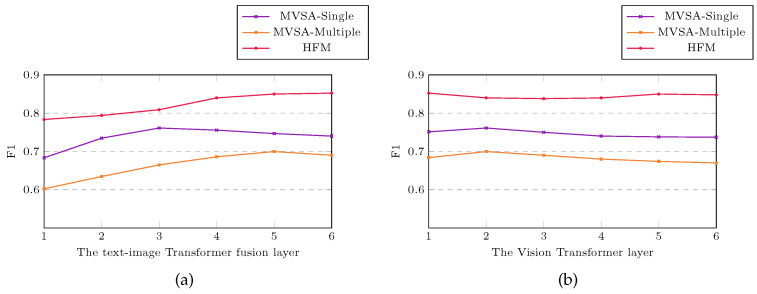
Experimental results of different layers of multi-layer fusion module. The solid line indicates the F1 score, while the x-axis denotes the layer count within the Transformer: (**a**) the text-image Transformer fusion layer; (**b**) the image Transformer layer.

To investigate the impact of different layers within the Transformer Encoder on the model’s performance, we conducted experiments by varying the number of layers for both the text-image Transformer bottleneck fusion and the image Transformer layers. This is illustrated in Figure 4, where (a) represents the variation of the text-image Transformer fusion layer from 1 to 6, and (b) represents the variation of the image Transformer layer from 1 to 6.

For our experiments, we considered different combinations of layers for the text-image Transformer fusion and image Transformer layers, such as 3-2, 5-2, and 6-1, respectively, for the three datasets. These combinations allowed us to analyze the contributions of text and images separately and understand their impact on the model’s performance.

Table 2 provides an overview of the results obtained from these experiments. Notably, our model relies more on text-based than image-based features in the HFM dataset. Consequently, we assign more Transformer layers to the text-related components within the multi-layer fusion (MLF) module, emphasizing their significance in capturing sentiment information effectively.

These experiments highlight the importance of considering the distinct contributions of text and images to the dataset. This allows us to optimize the model by appropriately allocating the Transformer layers within the MLF module to leverage each modality’s specific characteristics and relevance for sentiment analysis tasks.

### 4.8. The Effect of Contrastive Learning

To demonstrate the effectiveness of our proposed contrastive learning approach in facilitating the model’s understanding of sentiment-related common features from multimodal inputs, we conducted a visualization experiment using the MVSA-Single dataset. We employed dimensionality reduction techniques to visualize the data feature vectors from the final layer of our model. In this experiment, we utilized the t-SNE (t-distributed stochastic neighbor embedding) dimensionality reduction method to generate a two-dimensional feature vector, which we then visualized.

**Figure 5:**
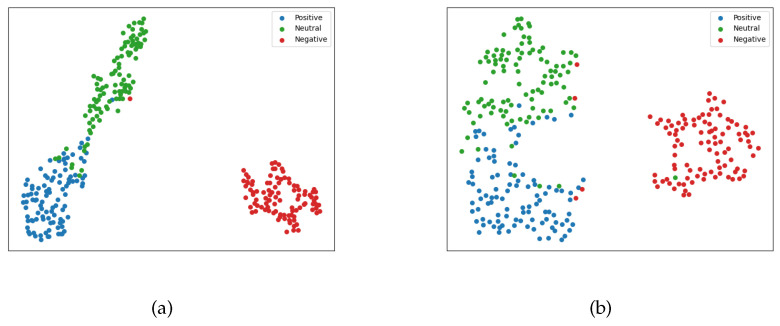
Cluster visualization of MVSA-Single: (**a**) supervised contrastive learning; (**b**) cross-entropy.

Figure 5a represents the visualization of the fusion result output from our model, while Figure 5b represents the visualization of the cross-entropy output from our model. The visualizations demonstrate that contrastive learning enhances the separation between positive and negative sentiments in the vector space, making the data aggregation patterns more discernible. This indicates that our model effectively distinguishes data points in the vector space based on shared characteristics among samples of the same emotional sentiment.

Furthermore, our visualization results exhibit a grouping pattern for the neutral sentiment data instead of scattering them across the vector space, as is observed with BERT. This is due to the relatively smaller amount of neutral sentiment data available. The grouping of neutral data indicates that our model captures common features associated with neutral sentiment, contributing to its improved performance.

Overall, these visualization results highlight the efficacy of incorporating contrastive learning in enabling the model to acquire and leverage common sentiment-related traits, thereby enhancing its overall performance.

### 4.9. Case Study

To provide a more intuitive understanding of our model’s validity, we present a set of illustrative examples and a case study demonstrating the model’s efficacy. In particular, we compare sentiment labels derived from our model and those predicted by the BERT model.

The case study is structured as follows: the leftmost column showcases the example image, the second column features the corresponding textual information, the third column displays the sentiment prediction determined by the BERT pre-trained model, and the final column exhibits our model’s performance. This layout is intended to facilitate a direct comparison between our model and the BERT pre-training model, thereby highlighting the relative merits of our approach.

As demonstrated in Table 4, relying solely on text-based sentiment analysis may lead to incorrect interpretations of users’ emotional inclinations. Take, for example, the first data point in Table 4. Although the text appears negative, adding a smiley face image introduces a positive sentiment. Similarly, the second data point’s text may initially suggest neutrality. However, the accompanying image conveys a negative sentiment, altering the overall emotional context. These examples underscore how effectively our model captures and processes multimodal information and the interactions between various modalities.

## 5. Conclusions

In conclusion, this paper introduces novel multimodal fusion methods for multimodal analysis tasks, specifically focusing on the association of individual statistical features across multiple modalities. Furthermore, we incorporate contrastive learning to aid the model in learning sentiment-related features from multimodal data and improve its ability to extract and fuse multimodal data features. Our proposed approaches have demonstrated superior performance through extensive experiments compared to baseline methods. These findings highlight the effectiveness of our methods in capturing and leveraging the synergies between different modalities, ultimately leading to improved performance in sentiment analysis tasks.

## Figures and Tables

**Table 1:** Number of data points for each sentiment category in each dataset.

Dataset	Label	Train	Val	Test
MVSA-Single	Positive	2147	268	268
	Neutral	376	47	47
	Negative	1088	135	135
MVSA-Multiple	Positive	9056	1131	1131
	Neutral	3528	440	440
	Negative	1040	129	129
HFM	Positive	8642	959	959
	Negative	11,174	1451	1450

**Table 2:** The experimental results.

Modality	Model	MVSA-Single		MVSA-Multiple		Model	HFM	
		Acc	F1	Acc	F1		Acc	F1
Text	CNN	0.6819	0.5590	0.6564	0.5766	CNN	0.8003	0.7532
	BiLSTM	0.7012	0.6506	0.6790	0.6790	BiLSTM	0.8190	0.7753
	BERT	0.7111	0.6970	0.6759	0.6624	BERT	0.8389	0.8326
	TGNN	0.7034	0.6594	0.6967	0.6180			
Image	ResNet-50	0.6467	0.6155	0.6188	0.6098	ResNet-50	0.7277	0.7138
	OSDA	0.6675	0.6651	0.6662	0.6623	ResNet-101	0.7248	0.7122
Multimodal	MultiSentiNet	0.6984	0.6984	0.6886	0.6811	Concat(2)	0.8103	0.7799
	HSAN	0.6988	0.6690	0.6796	0.6776	Concat(3)	0.8174	0.7874
	Co-MN-Hop6	0.7051	0.7001	0.6892	0.6883	MMSD	0.8344	0.8018
	MGNNS	0.7377	0.7270	0.7249	0.6934	D&R Net	0.8402	0.8060
	CLMLF	0.7533	0.7346	0.7200	0.6983	CLMLF	0.8543	0.8487
	Ours	**0.7689**	**0.7611**	**0.7352**	**0.6996**	Ours	**0.8663**	**0.8522**

**Table 3:** Ablation results of our model. “w/o Transformer” refers to a simple concatenation of text features and image features without using a Transformer model.

Network	MVSA-Single		MVSA-Multiple		HFM	
	Acc	F1	Acc	F1	Acc	F1
Ours	**0.7689**	**0.7611**	**0.7352**	**0.6996**	**0.8663**	**0.8522**
w/o Statistics	0.7569	0.7346	0.7234	0.6994	0.8634	0.8478
w/o Attention bottlenecks fusion	0.6951	0.6801	0.6829	0.6738	0.8012	0.7991
w/o Sup	0.7347	0.7212	0.7194	0.6834	0.8439	0.8011

**Table 4:** Example of data misclassified by BERT and correctly classified by Ours.

Image	Text	BERT	Ours
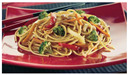	Sweet & Spicy Stir Fry	Neutral	Positive
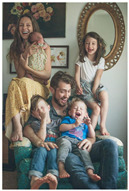	I really can see love, peace, and happiness in it	Neutral	Positive
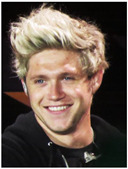	Niall onstage in Edmonton last night !!!	Negative	Positive

## Data Availability

Not applicable.
